# Response of Intestinal Bacterial Flora to the Long-Term Feeding of Aflatoxin B1 (AFB1) in Mice

**DOI:** 10.3390/toxins9100317

**Published:** 2017-10-12

**Authors:** Xiai Yang, Liangliang Liu, Jing Chen, Aiping Xiao

**Affiliations:** Institute of Bast Fiber Crops, Chinese Academy of Agricultural Sciences, Changsha 410205, China; yangxiai@caas.cn (X.Y.); liuliangliang@caas.cn (L.L.); 17673116054@163.com (J.C.); xap5@sina.com (A.X.)

**Keywords:** mice, aflatoxin B1, intestinal bacterial flora, response

## Abstract

In order to investigate the influence of aflatoxin B1 (AFB1) on intestinal bacterial flora, 24 Kunming mice (KM mice) were randomly placed into four groups, which were labeled as control, low-dose, medium-dose, and high-dose groups. They were fed intragastrically with 0.4 mL of 0 mg/L, 2.5 mg/L, 4 mg/L, or 10 mg/L of AFB1 solutions, twice a day for 2 months. The hypervariable region V3 + V4 on 16S rDNA of intestinal bacterial flora was sequenced by the use of a high-flux sequencing system on a Miseq Illumina platform; then, the obtained sequences were analyzed. The results showed that, when compared with the control group, both genera and phyla of intestinal bacteria in the three treatment groups decreased. About one third of the total genera and one half of the total phyla remained in the high-dose group. The dominant flora were *Lactobacillus* and *Bacteroides* in all groups. There were significant differences in the relative abundance of intestinal bacterial flora among groups. Most bacteria decreased as a whole from the control to the high-dose groups, but several beneficial and pathogenic bacterial species increased significantly with increasing dose of AFB1. Thus, the conclusion was that intragastric feeding with 2.5~10 mg/mL AFB1 for 2 months could decrease the majority of intestinal bacterial flora and induce the proliferation of some intestinal bacteria flora.

## 1. Introduction

Intestinal microflora play important roles in affecting the health of hosts through many aspects, including nutrition decomposition and transformation, immunity intrusion, biological antagonism and anti-aging [1,2]. Some degree of internal and external stimulation or interference of the body may trigger a change in the numbers or the components of intestinal microflora, cause physiochemical reactions, and lead to diseases [3,4]. Intestinal microflora were proved to be able to bind, transform, degrade, and transfer mycotoxins [5,6,7]. Some intestinal bacterial strains isolated from intestines and other environments were able to transform and degrade some mycotoxins in vitro [8,9,10]. Considering the functional influences, scientists attempted to find methods which could balance or optimize intestinal microflora communities to achieve normal or active levels in intestinal tracts and keep the body healthy. For instance, one could feed hosts with curing drugs, nutritional elements, probiotics or symbiotic foods, etc. To a large extent, the normality of intestinal microflora could symbolize the health level of the body [11,12,13].

Aflatoxins are highly hazardous contaminants in common food and feed, mainly originating from secondary metabolites in *Aspergillus flavus* and *Aspergillus parasiticus* [14,15]. Aflatoxin B1 (AFB1) is the predominant type in aflatoxins and was demonstrated to be highly toxic, mutagenic, teratogenic, and carcinogenic to many animals [16,17]. As a potent carcinogen, AFB1 reacts mainly with liver DNA and serum albumin in a dose-dependent manner [18]. Toxicity from AFB1 can lead to a reduction in production, hepatotoxicity, nephrotoxicity, disturbance in the gastrointestinal tract and reproduction, immune suppression, and disease susceptibility. Contamination of feed with aflatoxins, especially AFB1, is one of the major concerns in poultry industry [19,20]. Because of its high toxicity, strict regulations are placed on foods and feeds containing AFB1, on a world-wide basis [21].

Like most mycotoxins, AFB1 not only damages body organs directly but also disturbs the normal activities of intestinal microflora in animals [22,23]. While previous studies have mainly focused on its toxicology and binding and while some have also attempted to transform and remove AFB1 to decrease the toxicity in some poultry species [24,25], the influential mechanism underlying the effect of AFB1 on intestinal microflora has not been found so far. Based on this investigation, a continuous feeding procedure was performed with KM mice in this study by using AFB1 solutions with various concentrations, which was designed comprehensively according to the dose of chronic aflatoxicosis in mice [26,27,28,29]. The research results might lay a foundation for discovering whether the disturbance of intestinal microflora under the toxicity of AFB1 is related to liver cancer or for finding feasible ways to reduce the toxicity through modulating intestinal bacterial flora.

## 2. Results

### 2.1. Operational Taxonomic Units (OTUs) and Rarefaction Curves

**Figure 1:**
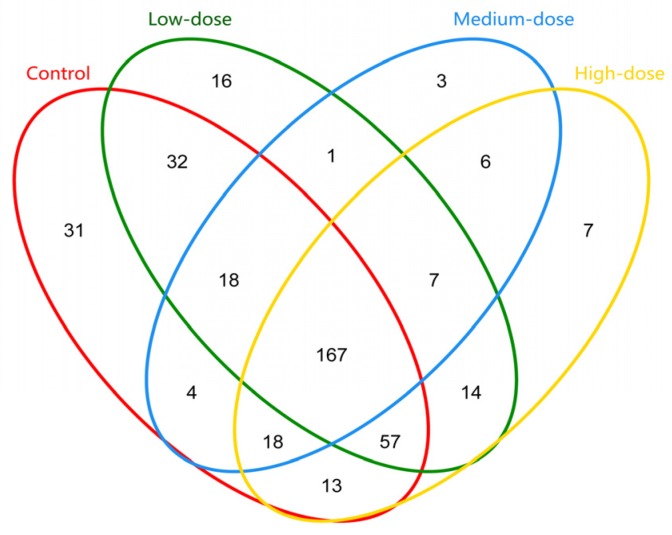
Venn map of OTU distribution in each group and between and among groups (97% similarity of OTUs). Different colors represent different samples. If, for instance, figure 100 was simultaneously marked in two different circles, this meant that the two samples had the same sequences categorized in the same OTUs, and the OTUs were 100.

The OTUs of each group and the overlapped OTUs among groups were drawn as a Venn picture [30]. The similarity of overlapped OTUs between or among groups was ≥97%. There were 167 OTUs contained in all groups, whereas the specific OTUs in the control, low, medium, and high dose groups were 31, 16, 3, and 7, respectively. Although the high-dose group had slightly higher OTUs than the medium-dose group, the OTUs in each group showed a decrease tendency as a whole (Figure 1).

**Figure 2:**
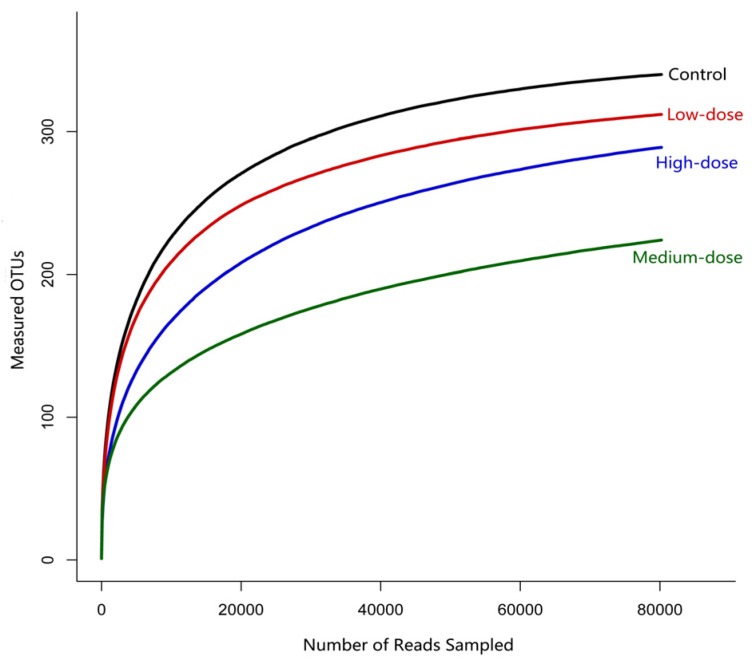
Rarefaction curves (97% similarity of OTUs). Horizontal ordinate: Sequences sampled randomly; Vertical ordinate: Measured OTUs. Rarefaction curves could estimate the depth of sequencing; the plateau indicates that the measured OTUs could reasonably represent the sampled sequences.

When the measured Operational Taxonomic Units (OTUs) [31] reached about 250, the rarefaction curves for the control and low-dose groups plateaued. However, when the measured OTUs were about 200 and 150, the rarefaction curves plateaued for the high- and medium-dose groups, respectively (Figure 2). All rarefaction curves of the four groups plateaued, which meant that the majority of sequences were involved in the analysis process for each group. Both OTUs and sequence numbers of the medium-dose group (less than 20,000 reads) were lower than those of the remaining groups (about 20,000 reads) based on Figure 2. The changing trend of the rarefaction curves was consistent with OTUs among groups.

### 2.2. Genera and Phyla Performances

**Figure 3:**
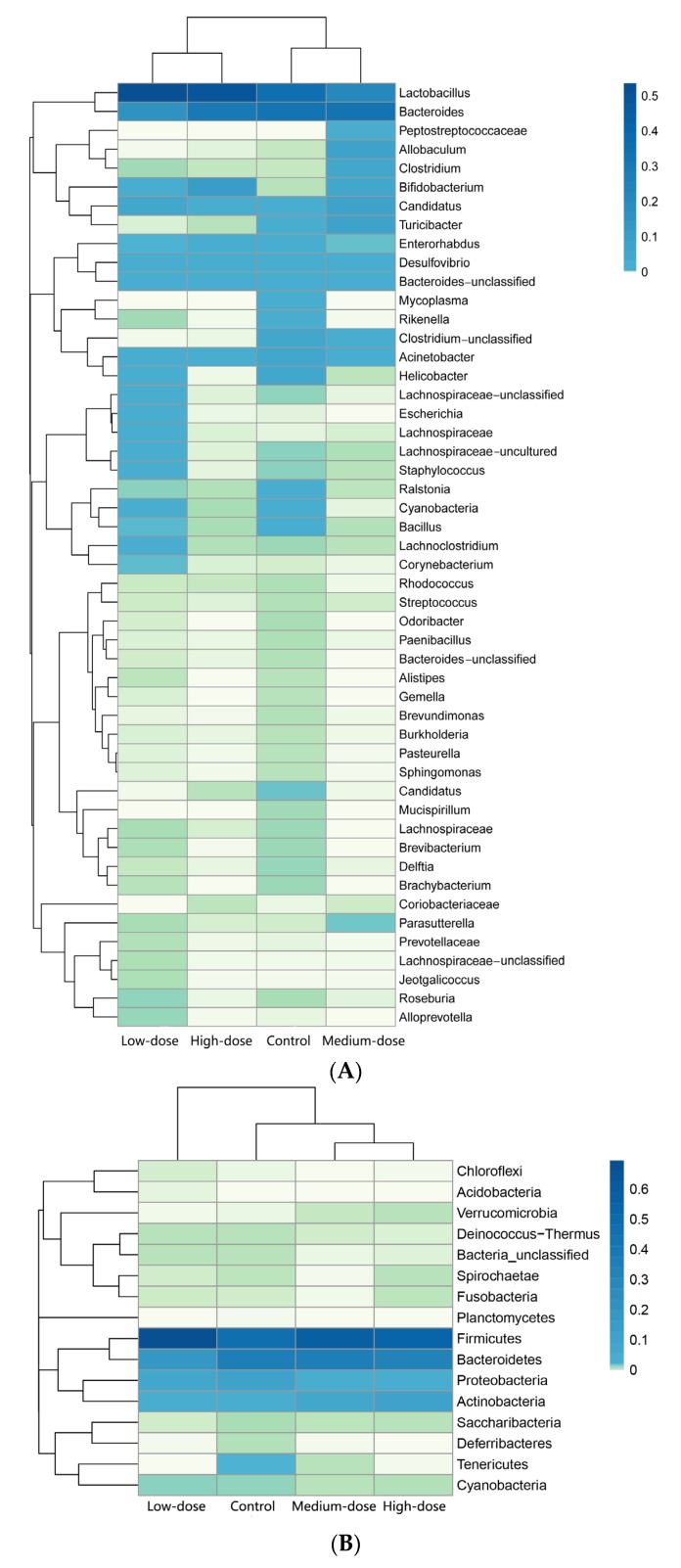
Heatmap of genus and phylum types and relative abundance of intestinal bacteria flora. (**A**) Heatmap of genus types; (**B**) Heatmap of phylum types. Different colors represent the relative abundances of different intestinal bacteria; the darker color indicates higher abundance.

The genus and phylum types and the relative abundances of intestinal bacteria were clustered as a Heatmap [32], where darker colors indicate a higher abundance of the bacterial flora. According to the Heatmap, the control and the low-dose groups had higher abundances and also more types of genera and phyla than the medium- and high-dose groups (Figure 3). The high-dose group had the fewest types of genera. Although there were more genera for both the control and low-dose groups, the genera types differed greatly between the two groups. The two genera with high abundance in the four groups were *Lactobacillus* and *Bacteroides*. The four genera with the second highest abundance in the four groups were *Candiatus*, *Desulfovibrio*, *Bacteroides* and *Acinetobacter* (Figure 3A). Except for the control group, which had an extra phylum *Tenericutes*, there were 4 phyla with high relative abundances in each group. These 4 phyla were *Firmicutes*, *Bacteroidetes*, *Proteobacteria* and *Actinobacteria*. There was no evident difference for each phylum among all the groups, and *Firmicutes* had the highest abundance in each group (Figure 3B).

**Figure 4:**
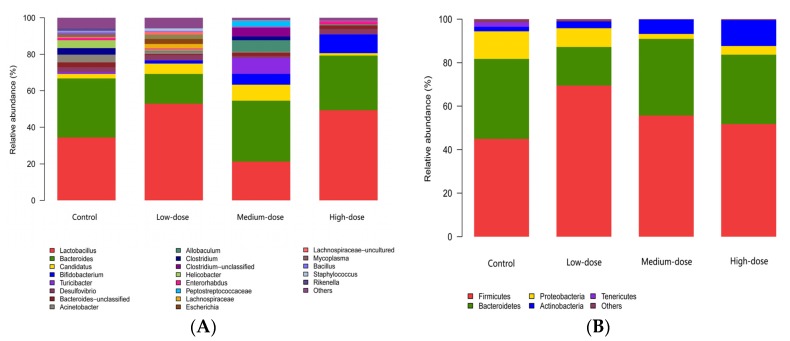
Column pictures of genus and phylum types and relative abundance of intestinal bacteria. Others: Intestinal bacterial flora with relative abundance <1% were included as others. (**A**) Column pictures of genera; (**B**) Column pictures of phyla. Different colors represent different intestinal bacteria; the percentage on the vertical ordinate indicates the relative abundance of intestinal bacteria.

The genus and phylum types and relative abundances of intestinal bacteria were drawn as column pictures [33]. These pictures contain similar information as in the Heatmap, showing that *Lactobacillus* and *Bacteroides* had high abundances in the four groups, while the other 4 genera mentioned in Figure 3 were also present as the second highest. There were visually fewer genera in the high-dose group compared with the rest of groups, and especially with respect to the control group. There was an interesting phenomenon that the abundance of *Lactobacillus* decreased, but the abundance of *Bacteroides* increased evidently in the medium-dose group, while the two genera increased, reaching a large proportion in the high-dose group that was nearly the same as in the control group (Figure 4A). With respect to the phyla, more than six phyla were present in the control and the low-dose groups, whereas only 4 phyla were present in the medium- and high-dose groups (Figure 4B).

### 2.3. Phylogenetic Tree

**Figure 5:**
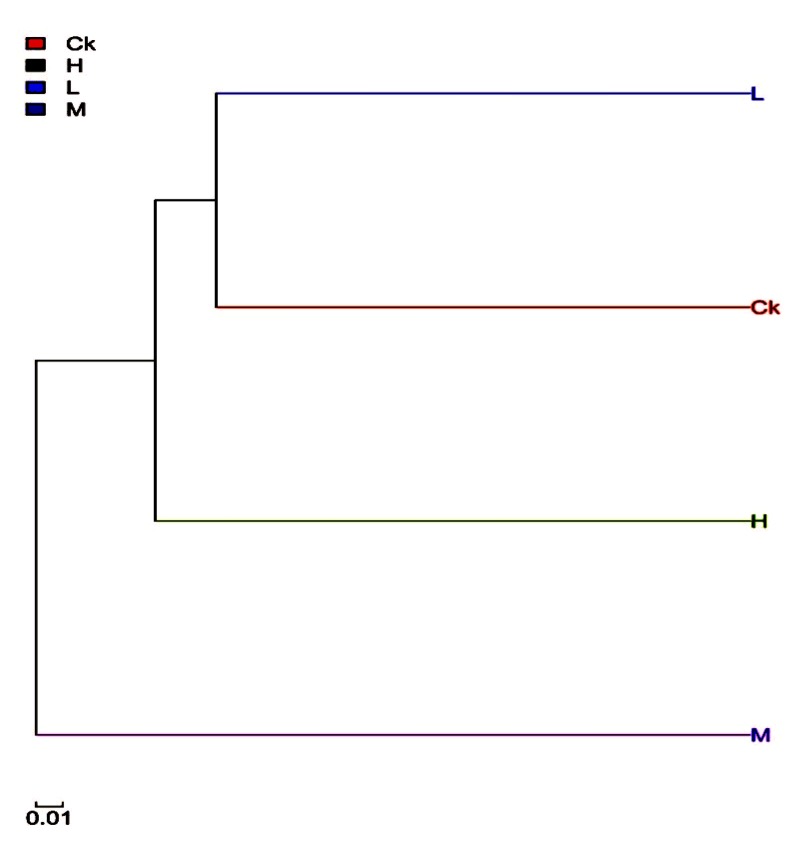
Phylogenetic clustering tree based on the Hierarchical clustering method. Branch size represents the genetic distance between samples; different colors represent different samples.

The phylogenetic tree was built based on the Hierarchical clustering method [34]. The intestinal bacteria of the control and the low-dose groups were clustered together (Figure 5), indicating that most bacteria in the two groups had a relatively close genetic relationship. The intestinal bacteria of the medium- and high-dose groups were clustered together, showing a close genetic relationship.

### 2.4. Differences in Relative Abundance of Bacterial Flora

**Figure 6:**
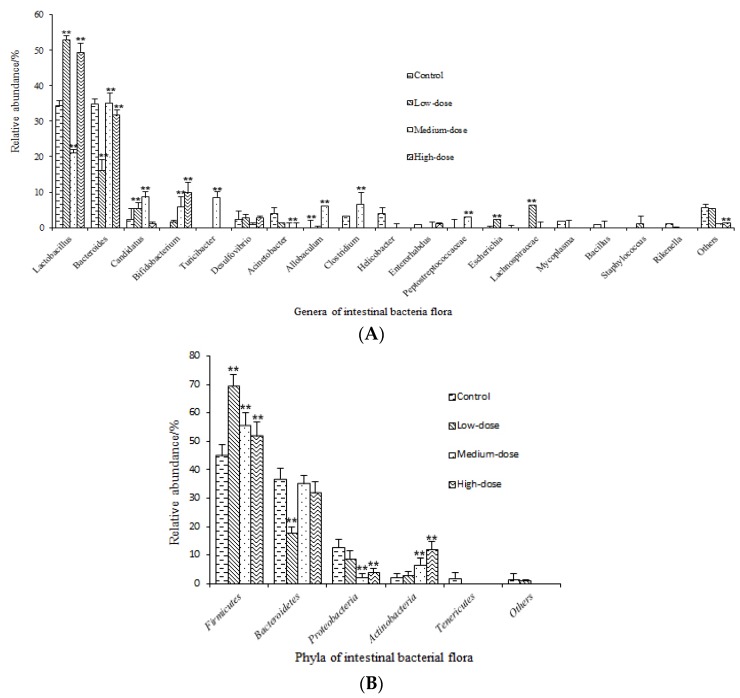
Difference in relative abundance of intestinal microflora between the control and the treatment groups. (**A**) Difference in relative abundance of intestinal microflora at the genus level; (**B**) Difference in relative abundance of intestinal microflora at the phylum level. ** means a significant difference (*p* < 0.01). Data were analyzed using one-way ANOVA. Difference between the control and the treatment groups was assessed by Duncan’s test.

The significance of the difference in the relative abundance of bacterial flora between groups is shown in Figure 6. Compared with the control, *Lactobacillus* increased significantly in the low-dose and the high-dose groups but decreased significantly in the medium-dose group. *Bacteroides* showed a significant decrease in the low-dose group and a slight decrease in the high-dose group, while no evident change occurred in the medium-dose group. *Bifidobacterium* showed a significant increase in the high-dose group. *Candidatus*, *Turicibacter*, *Allobaculum*, *Clostridium*, and *Peptostreptococcaceae* had a significant increase in the medium-dose group and then decreased dramatically in the high-dose group. *Escherichia* and *Lachnospiraceae* increased significantly in the low-dose group and then decreased dramatically in the medium-dose and the high-dose groups (Figure 6A). The majority of the intestinal bacterial flora at the genus level decreased as a whole, and the results in Figure 6 confirm the results in Figure 3 and Figure 4. At the phylum level, *Firmicutes* showed a significant increase in all treatment groups, *Bacteroidetes* showed a significant decrease in the low-dose group, while no evident change occurred in the other two treatment groups, and *Actinobacteria* showed an increase in all treatment groups, especially in the high-dose group. The remaining phyla showed a decreasing tendency from the control to the high-dose groups (Figure 6B). The statistical data of the relative abundance of intestinal microflora are shown in Table 1.

## 3. Discussion

Although knowledge of microflora in animal intestinal tracts is very limited, the view about their mediation and maintenance roles in host health is highly agreeable. Many important immune and metabolic disorders, including diabetes, obesity, behavioral disorders, and chronic inflammation, are known to be partially caused by the imbalance of interactions between host and intestinal microflora [35,36,37]. It is crucial to keep intestinal microflora in balance for host health. 

It is well accepted that the two dominant normal intestinal microflora in mammalians belong to the phyla *Firmicutes* and *Bacteroidetes*, which are strict anaerobic bacteria [38]. *Bacteroides*, *Lactobacillus*, *Streptococcus*, *Clostridium*, *Enterococcus*, *Bifidobacterium*, *Candidatus*, etc. are the main genera with a larger quantity of microflora which inhabit the intestines [39,40,41]. However, major mycotoxins including deoxynivalenol, zearalenone, ochratoxin A, fumonisin B1 and aflatoxin B1 are proven to be able to disturb the stability of mammalian intestinal microflora [42]. Feeding with a certain dose of deoxynivalenol for 4 weeks by oral gavage increased *Bacteroides* and *Prevotella* but decreased *Escherichia coli* in rat intestines [43]. Zearalenone, given by oral gavage, reduced the total cultivable aerobic bacteria in pig intestines [44]. Ochratoxin A enhanced the *Lactobacilliaceae* family, increased the facultative anaerobes and decreased the microbial α-diversity in rat intestines [45]. Fumonisin B1 changed the similarity between microbial CE-SSCP profiles in pigs after a fumonisin-based feeding [46].

In our study, both genus and phylum showed a decreasing tendency from the control to the high-dose group as a whole, which was consistent with the finding that there was a reduction in microbial diversity in the colon from the healthy rats exposed chronically to AFB1 for 4 weeks [47]. There were about 8 genera with higher relative abundances in the high-dose group, which contained the least genera among all the groups (Figure 3A). Compared with the control group, the high-dose group had about one third of the total genera and nearly half of the total microflora (Figure 4A). There were about 4 phyla in both the medium-dose and the high-dose groups (Figure 4B). Compared with the control group, the high-dose group had about one half of the total phyla. The statistical analysis showed the same decreasing tendency for most bacterial flora except for several special ones (Table 1, Figure 6). However, a chronic exposure to AFB1 could not change the proportion of *Firmicutes* and *Bacteroidetes* [47].

The two dominant flora in mice were *Lactobacillus* and *Bacteroides*. However, their relative abundances were different among the groups. This observation might be explained that AFB1 with different doses disturbed the two dominant bacteria and led to different results. These two dominant bacteria showed similar total proportions in the control, the low-dose, and the high-dose groups but displayed the lowest proportion in the medium-dose group (Figure 4). Different abundance with a similar total proportion under different AFB1 doses meant that these two types of bacteria finally recovered to the normal proportion, even though they were fed with AFB1 at a high dose for 2 months. They showed strong viabilities, indicating that the two bacteria were tolerant and adaptable to a certain dose of AFB1. Wang et al. also found that different bacterial flora had different tolerances to AFB1, three *Clostridiales* species had the largest increase while 2 *Lactobacillales* species had the largest decrease with increasing AFB1 dose in their study [47]. Using a cultural method, Galarza-Seeber et al. discovered that the facultative anaerobe (coliforms) population was 10-fold higher than the control in cecum of broilers exposed to AFB1, whereas there was only a numerical non-significant rise observed for other microbial populations [48].

There were five genera with high relative abundances in the medium-dose group but not in other 3 groups, especially not in the high-dose group. They were *Peptostreptococcaceae*, *Allobaculum*, *Clostridium*, *Turicibacter*, and *Cadidatus* (Figure 3A and Figure 6A). Among these genera, *Clostridium* contains several significant human pathogens, including the causative agent of botulism and an important cause of diarrhea [49,50]. A high-fat diet can lead to the expansion of the cluster XI of genus *Clostridium*, which could produce secondary bile acid, causing a phenotypic change in hepatic stellate cells to secrete proinflammatory cytokines, and eventually resulting in hepatocellular carcinoma in mice [51]. *Turicibacter* spp. has been strongly associated with immune function and bowel disease [52]. The proportion of *Turicibacter* was significantly more abundant in Inflammatory Colorectal Polyps (ICRPs)-affected in miniature dachshunds [53]. The high abundances of these bacteria may have resulted from the animals been maintained under unhealthy conditions. However, all these genera were not found in the high-dose group. The mechanism needs to be further explored to understand whether a high dose of AFB1 killed these genera or other factors led to the phenomenon.

Since there are only two research papers published that are relevant to the influence of AFB1 on gut microflora [47,48], more studies are required.

## 4. Conclusions

In conclusion, the intestinal bacterial flora in mice could be strongly disturbed by intragastric feeding with AFB1 solutions ranging from 2.5 mg/L to 10.0 mg/L for 2 months. *Lactobacillus* and *Bacteroides* were the two dominant bacterial flora, which could be induced to the same level as the control group under a high dose of AFB1 in this study. Nearly two thirds of the total genera and two more phyla finally disappeared. There might be several tolerant, adaptable, and inducible bacterial flora in mouse intestines under a certain dose of AFB1, but this possibility needs be investigated further.

## 5. Materials and Methods

### 5.1. Diet Information

With no antibiotics, hormones, and preservatives, the food ingredients were 27.0% corn starch, 19.0% wheat bran, 16% rice starch, 16.0% soybean dreg, 13.0% fish powder, 3.0% bone powder, 2.3% yeast powder, 0.5% salt, 0.1% compound vitamin and 0.1% trace elements; sterile water was added to ensure a 10% water content. After being mixed evenly, the mixture was sterilized at 121 °C for 20 min in a high pressure steam sterilizer.

### 5.2. AFB1 Solutions Preparation

The AFB1 stock solution was prepared by dissolving 0.01g AFBI powder (99.9% of purity, Solarbio Company, Beijing, China) in 500 mL of 2% sterile aqueous ethanol. The solution was stirred with a magnet stirring bar at 150 rpm under 50 °C for 30 min and then diluted with sterile water to reach the concentrations of 2.5 mg/L, 4 mg/L, and 10 mg/L. 

### 5.3. Animal Trial

All animal experiments were approved by the Animal Care and Use Committee of Institute of Bast Fiber Crops, Chinese Academy of Agricultural Sciences (Changsha, Hunan). A total of 24 KM mice (SPF grade, Silaida Co., Ltd.. Hunan, China) with no specific pathogens were used in this study. Their average weight was about 20 ± 2 g. Equal numbers of males and females were adaptively fed in a quiet environment at 24 °C temperature and 65% humidity. The mice were then randomly divided into 4 groups, which were labeled as the control, low-dose, medium-dose, and high-dose groups. Each group was consisted of an equal number of males and females. The control group was fed intragastrically with sterile water, while the low-dose, the medium-dose, and the high-dose groups were fed intragastrically with 2.5 mg/L, 4 mg/L, and 10 mg/L of AFB1 solutions, respectively. The feeding dosage was 0.4 mL per mouse each time and 2 times a day for 2 months. Other conventional food was administered normally.

### 5.4. Sample Collection

The animals were killed by cervical dislocation and dissected aseptically on a laminar flow bench after the feeding treatment was over. Small intestinal contents (from jejunum to rectum) were collected from 2 randomly chosen mice (one male and one female) in each group, then stirred evenly with a sterile glass rod under a sterile environment, and considered as one replicate. Three replicates were used to analyze intestinal bacteria.

### 5.5. Intestinal Bacterial Flora Information

The total DNA was extracted from the obtained intestinal contents according to the instruction of the FastDNA^®^ Spin Kit for Feces (MP Company, CA, USA) and then tested by electrophoresis on a 1% agarose gel. After being recycled and purified with AxyPrepDNA (Axygen Company, CA, USA), the bands were amplified with primers 338F: ACTCCTACGGGAGGCAGCA and 806R: GGACTACHVGGGTWTCTAAT, which were specifically designed for testing the hypervariable region V3 + V4 zones of intestinal bacteria DNA. The PCR reaction mixture contained 4 μL of 5 × Fast Pfu buffer, 2 µL of 2.5 mM dNTPs, 0.8 µL of 5 µM Forward Primer, 0.8 µL of 5 µM Reverse Primer, 0.4 µL FastPfu Polymerase, 0.2 µL BSA, and 10 ng Template DNA. Dd H_2_O was added to reach the final volume to 20 µL. The reaction conditions as follows: an initial denaturing cycle at 95 °C for 3 min, with 28 cycles, denaturation at 95 °C for 30 s, annealing at 55 °C for 30 s, and extension at 72 °C for 45 s, with a final extension step at 72 °C for 10 min. Electrophoresis on 2% agarose gels was used to test the PCR products, the gene library was built, and the sequences were analyzed by Frasergene company (Wuhan, Hubei, China) with the Miseq Illumina System.

### 5.6. Statistical Analysis

SPSS software 22.0 (IBM Corp, Armonk, NY, USA) was used to analyze the significance of differences in the relative abundances of intestinal bacterial flora between groups.

## Figures and Tables

**Table 1:** Relative abundance of intestinal microflora in mice treated with different doses of AFB1.

Taxon	Control	Low-Dose	Medium-Dose	High-Dose
*Lactobacillus*	34.45 ± 0.69	52.99 ± 1.91 **	21.16 ± 2.01 **	49.40 ± 2.20
*Bacteroides*	32.32 ± 1.86	16.27 ± 1.64 **	33.36 ± 2.21	29.91 ± 1.80
*Candidatus*	2.52 ± 0.10	5.65 ± 0.13 **	8.80 ± 0.14 **	1.32 ± 0.05
*Bifidobacterium*	0.11 ± 0.04	1.71 ± 0.15	5.98 ± 0.42 **	10.19 ± 0.75 **
*Turicibacter*	0.96 ± 0.06	0.05 ± 0.01	8.70 ± 0.33 **	0.11 ± 0.03
*Desulfovibrio*	2.57 ± 0.11	2.97 ± 0.12	1.07 ± 0.05	2.89 ± 0.14
*Bacteroides-unclassified*	2.63 ± 0.10	0.57 ± 0.05	1.83 ± 0.06	1.98 ± 0.10 **
*Acinetobacter*	4.20 ± 0.01	1.56 ± 0.01	0.55 ± 0.01 **	0.66 ± 0.01 **
*Allobaculum*	0.07 ± 0.00	0.08 ± 0.00	6.20 ± 0.20 **	0.03 ± 0.00
*Clostridiium*	3.52 ± 0.08	0.01 ± 0.00	4.83 ± 0.05 **	0.02 ± 0.00 **
*Helicobacter*	4.23 ± 0.15	0.66 ± 0.01	0.08 ± 0.00	0.01 ± 0.00 **
*Enterorhabdus*	1.15 ± 0.10	0.54 ± 0.02	0.43 ± 0.02	1.43 ± 0.04
*Peptostreptococcaceae-unclassified*	0.00 ± 0.00	0.00 ± 0.00	3.22 ± 0.50 **	0.00 ± 0.00
*Lachnospiraceae-unclassified*	0.26 ± 0.01	2.52 ± 0.07	0.03 ± 0.00	0.04 ± 0.01
*Escherichia*	0.03 ± 0.01	2.62 ± 0.05 **	1.25 ± 0.02	0.02 ± 0.00
*Lachnospiraceae*	0.03 ± 0.00	2.36 ± 0.12 **	0.05 ± 0.01	0.04 ± 0.00
*Lachnospiraceae-uncultured*	0.30 ± 0.01	1.64 ± 0.02	0.14 ± 0.01	0.04 ± 0.00
*Mycoplasma*	2.03 ± 0.07	0.00 ± 0.00	0.00 ± 0.00	0.00 ± 0.00
*Bacillus*	1.04 ± 0.07	0.48 ± 0.02	0.12 ± 0.01	0.17 ± 0.00
*Staphylococcus*	0.29 ± 0.00	1.38 ± 0.12	0.09 ± 0.00	0.03 ± 0.00
*Rikenella*	1.31 ± 0.05	0.18 ± 0.01	0.02 ± 0.00	0.05 ± 0.00 **
Others	05.90 ± 0.17	5.63 ± 0.15	1.32 ± 0.05 **	1.6 ± 0.08 **

Each value is the mean ± standard deviation (*n* = 3). Data were analyzed using one-way ANOVA. Differences between control and treatment groups were assessed by Duncan’s test. ** means values are significantly different (*p* < 0.01).
